# Adaptation reveals sensory and decision components in the visual estimation of locomotion speed

**DOI:** 10.1038/s41598-018-30230-1

**Published:** 2018-08-29

**Authors:** George Mather, Todd Parsons

**Affiliations:** School of Psychology, University of Lincoln, Lincoln, UK

## Abstract

Locomotion speed provides important social information about an individual’s fitness, mood and intent. Visual estimation of locomotion speed is a complex task for the visual system because viewing distance must be taken into account, and the estimate has to be calibrated by recent experience of typical speeds. Little is known about how locomotion speed judgements are made. Previous research indicates that the human visual system possesses neurons that respond specifically to moving human forms. This research used point-light walker (PLW) displays that are known to activate these cells, in order to investigate the process mediating locomotion speed judgements. The results of three adaptation experiments show that these judgements involve both a low-level sensory component and a high-level decision component. A simple theoretical scheme is proposed, in which neurons sensitive to image flicker rate (temporal frequency) provide a sensory speed code, and a benchmark ‘norm’ value of the speed code, based on prevailing locomotion speeds, is used to make decisions about objective speed. The output of a simple computational model of the scheme successfully captured variations in locomotion speed in the stimuli used in the experiments. The theory offers a biologically-motivated account of how locomotion speed can be visually estimated.

## Introduction

Predators are known to use the movement speed of other animals as a basis for prey selection during chases, preferring slower targets^1–3^. Vulnerability to attack in humans is also related to locomotion kinematics^4,5^. Human walking speed varies with the walker’s mood, temperament and intent, and thus provides important social information about the walker’s physical and mental state^6,7^. For example, people walk faster when they are happy than when they are sad. However, it is not a simple task for the visual system to estimate an individual’s walking pace as they move across the field of view. Firstly, the projected retinal speed of a walking figure varies inversely with viewing distance (retinal speed halves with each doubling of viewing distance). The response of neurons in the early parts of the visual system varies with retinal speed^8^, so estimation of locomotion speed independent of viewing distance must involve a computation of objective speed that copes with variations in retinal speed. Secondly, and even if this problem is solved, there is no single objective speed that is the typical or ‘natural’ speed at which humans walk (or run), against which one can reliably infer intent, mood, fitness and so on. For example, people in the UK and Netherlands have been reported to walk 50% faster on average than people in Brazil and China^9^. So judgements of locomotion speed need to be calibrated on the basis of recent experience.

Consistent with this need for calibration, it was recently reported that judgements of locomotion speed are adaptable^10^. After viewing a video depicting slowed-down or speeded-up human locomotion (recordings of people walking along a high street) for a short time, experimental participants made judgements about the locomotion speed depicted in brief test videos of the same scene. The results showed that apparently ‘natural’ or ‘normal’ locomotion speed shifted towards the speed of the previously viewed stimulus; it was slower after adapting to slowed-down locomotion, and faster after adapting to speeded-up locomotion. This shift in perceived locomotion speed is a form of normalisation to recently experienced stimuli, similar to other normalisation effects in the perception of faces, blur and colour^11–13^, indicating that judgements of an object’s speed are indeed calibrated on the basis of prevailing speeds in recent experience.

Little is known about how the visual system estimates objective speed from the retinal speed signals supplied by the eye. Multiple centres in the visual cortex contain neurons that respond to the direction and speed of retinal movement^14^. None have yet been found that respond to objective speed. An extrastriate cortical area relatively late in the processing hierarchy, located in temporal cortex on the superior temporal sulcus (hence called area STS) is involved in the perception of moving human forms^15,16^, and is thus a prime candidate area for processing locomotion speed signals. ‘Point-light walker’ (PLW) displays have become established as a stimulus paradigm for targeting neural systems that process locomotion. PLW stimuli depict locomoting forms by means of only a dozen isolated points of light located at the major joints of the figure. A number of different animals are known to be sensitive to PLWs, including macaques, marmosets, cats, dogs, rats, pigeons, chicks, and fish^17–23^. Human neuroimaging studies using PLWs have identified STS as an area that is specialised for the analysis of human locomotion^24^.

**Figure 1:**
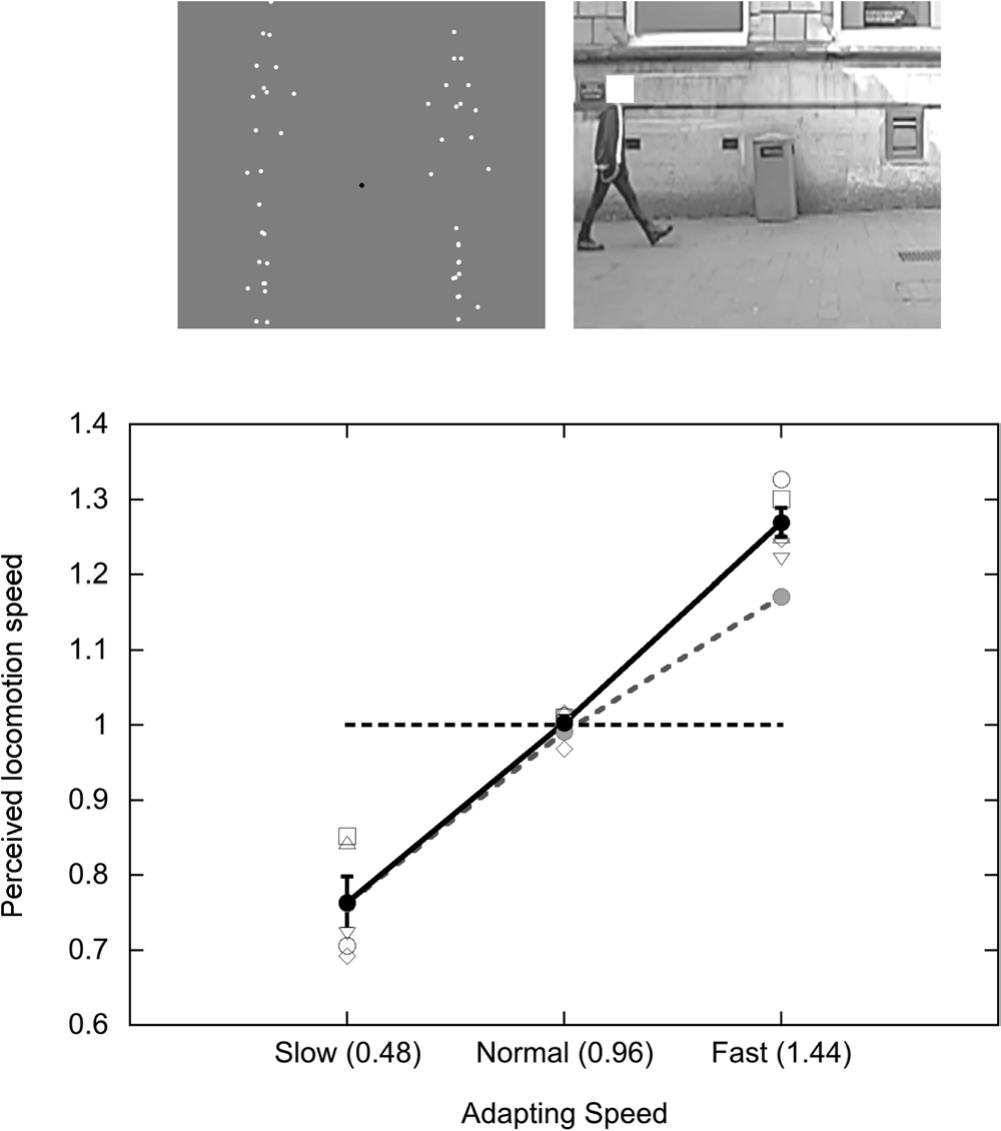
Stimuli and results of Experiment 1. Top: Still frames from the stimuli used in the experiments. The left-hand image shows an example frame from the PLW animation. Each of the four PLWs varied from trial to trial in its position, facing direction, size and step cycle phase. The right-hand image shows a frame from the full-cue walking video, recorded in the local high street in Lincoln (the face has been obscured in the Figure to avoid identification, but was visible in the experiment). Both stimuli were 512 × 512 pixels, surrounded by a uniform grey background (same luminance as in the PLW display). Bottom: Results of experiment 1. The solid black line and circles show mean apparent PLW locomotion speed in three adapting conditions (slow, normal, fast), +/−1 SE. The open symbols show results from individual participants. The grey dotted line and symbols shows the mean apparent walking speed in the corresponding experiment^10^ using full-cue videos. The black dashed horizontal line shows normal speed (1x playback).

A set of three psychophysical experiments used PLW displays to study the processes that mediate calibration of locomotion speed judgements. The first experiment tested whether PLW displays are effective as a stimulus for driving adaptation to locomotion speed, as predicted if previously identified neural systems responsive to human locomotion are involved. Participants adapted to a display containing a set of four PLWs all walking at one of three locomotion speeds (slow, natural or fast) for 30 seconds, and then made ‘slow’ versus ‘fast’ speed judgements of brief (500 ms) test displays containing four PLWs all walking at one of a range of possible speeds. ‘Natural’ speed was established using a pilot experiment. The four PLW figures in each adapting and test display varied in size, position, and facing direction (while walking at the same locomotion speed; see still-frame at top-left in Fig. 1) so as to dissociate the local retinal speed of each dot in the PLWs from the speed of the locomotion itself. Any adaptation effects would therefore reflect neural processes that do not respond simply to retinal speed.

## Methods

### Ethics and participants

All experiments were performed in accordance with institutional guidelines and regulations. The experimental protocol was approved by the School of Psychology Ethics Committee, University of Lincoln, UK. All participants gave their informed consent to take part in the experiments. Five participants took part in each experiment, four naïve and one author. Naïve participants were drawn from a pool of twelve volunteers. All participants had normal or corrected-to-normal vision.

### Apparatus

Stimuli were presented on a ViewPixx 3D Lite (VPixx, QC, Canada) flat panel display monitor, which has a spatial resolution of 1920 × 1080 pixels, a refresh rate of 120 Hz and screen width of 52 cm. The monitor was gamma-corrected using a LS100 luminance meter (Minolta, Osaka, Japan). A chin rest ensured that each participant viewed stimuli from a constant distance of 300 cm (0.0052° per pixel). Stimulus presentation and data collection were controlled by Psychtoolbox for Matlab^25,26^ (The Mathworks Inc., Natick, MA), running on a Dell Windows PC.

### Stimuli

All PLW displays contained four PLW figures defined by light dots (luminance 103 cd/m^2^) against a uniform grey background (luminance 20 cd/m^2^). One PLW figure was placed in each of the four quadrants of an invisible square (512 × 512 pixels, 2.64 × 2.64 deg) around a small central fixation marker. Each figure was defined by thirteen dots (two dots for the ankles, knees, hips, wrists, elbows, and shoulders; and one dot for the head). The diameter of each dot was 6 pixels or 0.031 deg. All figures were presented in sagittal view, walking sideways as if on a treadmill. The locomotion of each figure was generated using a standard algorithm for PLWs^27^, in which a single step cycle (two strides) is sampled as a sequence of animation frames. A pilot experiment established that an animation containing 152 movie frames per step cycle (at an animation rate of 120 Hz) appeared to be a natural walking pace to naïve observers. This corresponds to a walking speed of 1.27 seconds per step cycle (very close to the value of 1.2 seconds used in the original algorithm). During the experiments, locomotion speeds that were slower or faster than this natural pace were created by varying the number of animation frames per step cycle. Slow adapting stimuli contained 317 frames per step cycle (2.64 s/step cycle, or 0.48x natural speed); fast adapting stimuli contained 106 frames per step cycle (0.88 s/step cycle or 1.48x natural speed); natural speed adapting stimuli contained 158 frames per step cycle (0.96x natural speed). These adapting speeds were used because they matched those used previously^10^, so data could be compared across the studies. A range of test locomotion speeds was created in the same way.

Several characteristics of the four PLW figures in each display varied randomly from presentation to presentation, making each adapting and test stimulus unique in several respects. The facing direction of the figure in each quadrant (left versus right) was selected randomly in each trial, independently of the figures in the other quadrants. Similarly, the torso height of each figure varied randomly from trial to trial between 20 and 80 pixels (0.1–0.44 deg). The stride length of each figure was a fixed proportion of that figure’s height (0.4x as defined in the algorithm). The *x* and *y* positions of each figure in its quadrant also varied randomly from to trial by a maximum distance corresponding to 25% of its height. In each trial all four PLW figures walked at the same locomotion speed, though their step cycles were randomly out of phase. So in any one presentation of the display, the average local (retinal) speed of the twelve dots defining each of the PLWs could vary by a factor of four even though all the walkers were animated at the same walking speed (dot speed also varied markedly within each walker of course, dependent on the dot’s location on the body). Any effect of adaptation to locomotion speed could not be attributed simply to the local retinal speed of each dot. Finally, the dots also varied in their retinal location because figure positions shifted randomly between presentations. Figure 1 (top-left) shows an example frame from the PLW display, in terms of the 512 × 512 pixel square within which PLWs were drawn. In this example the top-left PLW faces right and the other PLWs face left.

The full-cue video displays used in Experiment 2 were the same as those used in Experiment 1 of a previous study^10^, namely recordings of people walking along the High Street in Lincoln. The camera was fixed in position on a tripod approximately perpendicular to the path of the walking figures. The recordings were made at 125 frames per second at a resolution of 720 × 576 pixels, and captured anyone who happened to be walking past the camera at the time the recording was made, so leftward and rightward walking directions varied randomly but were approximately balanced. Videos were cropped to 512 × 512 pixels prior to display, and thus occupied the same visual angle and location on the display screen as the PLW display (see top of Fig. 1). Locomotion speed in the videos was controlled during playback by varying the presentation duration of each video frame in the sequence (1–4 display refreshes), and the frame offset between successively presented frames (offset between 1 and 5 frames).

### Design and Procedure

All experimental procedures were approved by the School of Psychology Ethics Committee, University of Lincoln, UK. All three experiments involved three adapting conditions (adaptation to slow, natural, or fast PLW locomotion speeds) run in separate sessions. The order of the adapting conditions was randomised across sessions and participants. Each session involved a 30-second period of exposure to an adapting PLW display followed by a test/top-up cycle involving 140 test trials. Each pass through the test/top-up cycle involved the following sequence:

1. ISI (grey screen) for 0.5 seconds
2. Test display for 0.5 seconds per display
3. ISI (grey screen) for 0.5 seconds plus the time taken for the participant to respond
4. Top-up adapting display for 6 seconds

(repeat until all trials completed).

The ISIs signposted the test displays, so that participants could make their response after the disappearance of the test display.

Experiments 1 and 2 used the Method of Single Stimuli (MSS): a single test display was presented (PLW display in Experiment 1, or full-cue video in Experiment 2) and the participant’s task was to report whether the locomotion speed of the walking figures in the test display was slower than natural pace or faster than natural pace, by pressing one of two buttons on a response pad. The locomotion speed of each test display was selected randomly from a range of seven possible values between 0.5x and 1.5x natural speed, so that by the end of each session the participant had accumulated twenty responses at each test speed. A cumulative normal function was fitted to the data obtained in each session (as defined in the Palamedes toolbox; http://www.palamedestoolbox.org) in order to estimate the mean of the distribution (50% point or P50), which corresponded to the test locomotion speed that appeared natural.

Experiment 3 used a two-alternative forced choice (2AFC) psychophysical procedure for estimating P50 values in a way that would not be influenced by decision bias. Two probe PLW stimuli were presented in each test trial, and the participant reported which probe appeared to move at a speed that was closer to natural. Further details on the procedure and P50 calculations are provided in Supplementary Information. As in Experiment 1, different adapting speeds were presented in different experimental sessions (0.48, 0.96, 1.44), with order randomized across participants.

### Data access

Individual participant data and model output is available as an Excel spreadsheet in an Open Science Framework project at: osf.io/cjkyt.

## Results

### Experiment 1

This experiment involved PLW displays as both adapting and test stimuli. Figure 1 plots apparently natural test speed (P50) as a function of the three adapting speeds (slow, natural and fast). The solid black line and circles represent the mean across the five observers. Each open symbol represents data from a specific participant. The dashed black line shows normal speed (1×); data should fall along this line if there is no effect of adaptation. The effect of adapting speed was highly significant in ANOVA (F(2,8) = 142.57; p < 0.0001; effect size η^2^_p_ = 0.97). For comparison, the broken grey line and symbols re-plot mean data from an earlier study^10^, in which adapting and test displays were video clips of people walking along the local High Street (see still-frame at top-right in Fig. 1), played back at the same speeds as used those in the present experiment.

The results show that the PLW display is very effective as a stimulus for driving adaptation to locomotion speed, despite the measures taken to minimise any contribution from local retinal speed adaptation. The pattern of results is consistent with an explanation in terms of normalisation to locomotion speed: Adaptation to natural speed produced no shift in perceived locomotion speed, whereas adaptation to slow or fast locomotion resulted in perceptual shifts towards the adapting speed. The PLW effect is at least as strong as that obtained using full-cue videos of real-life locomotion, despite the highly impoverished display (only thirteen points per walker). Averaged across the fast and slow adapting conditions, the shift in P50 relative to natural speed adaptation is actually slightly larger for PLW displays (25.3%) than for video displays (20.47%).

If both video and PLW adaptation effects are mediated by the same neural process, then the adaptation should transfer across the two stimulus types. Experiment 2 tested for cross-adaptation between PLW and video displays. Participants adapted to PLW displays (same stimuli and adapting speeds as in Experiment 1) and were tested using full-cue videos (MSS as in Experiment 1).

### Experiment 2

**Figure 2:**
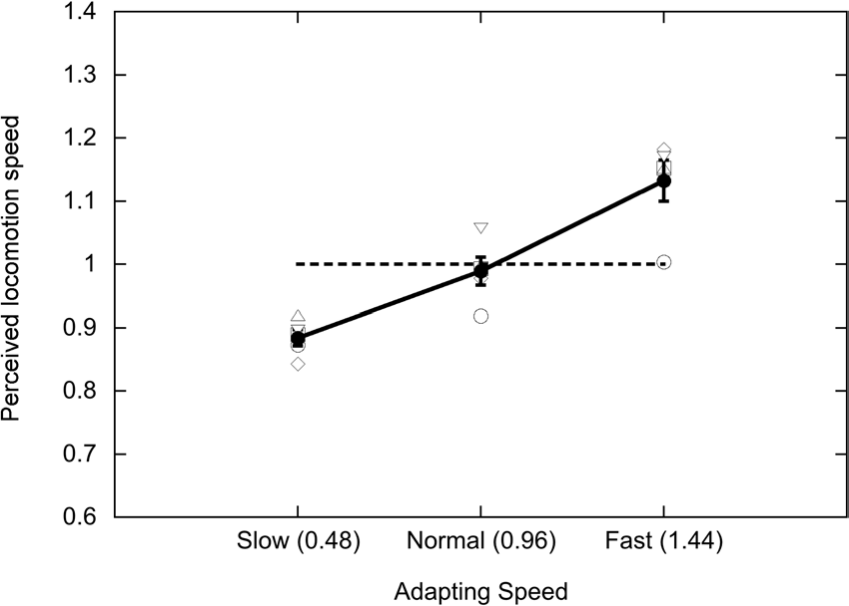
Results of Experiment 2. Apparent natural test speed as a function of adapting speed, following cross-adaptation between PLW adapting stimuli and full-cue video test stimuli. Conventions are the same as those in Fig. 1.

Figure 2 plots apparent natural test speed as a function of adapting speed, using the same conventions as those in Fig. 1. The effect of adapting speed was highly significant in ANOVA (F(2,8) = 46.73; p < 0.0001; effect size η^2^_p_ = 0.92). The results of Experiment 2 show that adaptation to the locomotion speed of PLWs influences later judgements of the locomotion speed of real figures recorded in videos. This result is remarkable given that there are many differences in the low-level visual properties of the two displays. However, the average P50 shifts are much smaller (12.4%) in the cross-adaptation experiment than in the uncrossed-adaptation experiment (25.3% for PLWs and 20.47% for videos; note that the vertical axes of Figs 1 and 2 are the same). The synthetic PLWs generated by the algorithm lack some of the details of human gait that would be present in videos of real humans, and this dissimilarity between adapting and test displays may have contributed to the smaller effect in the cross-adaptation experiment.

In each test trial of Experiments 1 and 2, the participant was shown a single stimulus containing walking figures, and was asked to report whether the figures appeared to walk faster or slower than a natural pace. It is well known that this psychophysical method (MSS) cannot distinguish between shifts in measured P50 values caused by sensory factors and those caused by decision factors^28–30^. Sensory shifts in P50 values reflect changes in the visual system’s response to stimuli. P50 shifts caused by decision factors reflect changes in the participant’s tendency to make one response rather than another regardless of the current state of their sensory system. For example, if the observer had a slight bias in favour of responding ‘faster’ regardless of which stimulus was presented, this would manifest itself in the data as a shift in the measured P50 towards a slower speed that would be indistinguishable from one due to sensory changes^28^. This kind of decision shift could occur for a variety of reasons, such as expectations, prior knowledge, or the semantic content of the stimuli^31^.

Psychophysical experiments rarely attempt to tease apart the contributions of sensory versus decision factors in P50 shifts (techniques based on Signal Detection Theory are not useful in this regard^32^). Experiment 3 was designed specifically to test the contribution of decision factors to the P50 shifts found in the first two experiments. It employed a variant of two-alternative forced-choice (2AFC) methodology^33^. A series of probe trials was presented after the initial phase of adaptation to PLW displays. Each probe trial involved the sequential presentation of two PLW displays depicting different locomotion speeds, drawn from a set of 15 possible pairs of displays. The task of the participant was to select the display (first or second) in which the locomotion speed was closer to a natural pace. As described in Supplementary Information, this procedure provides a way to estimate P50 that is not influenced by decision bias.

### Experiment 3

**Figure 3:**
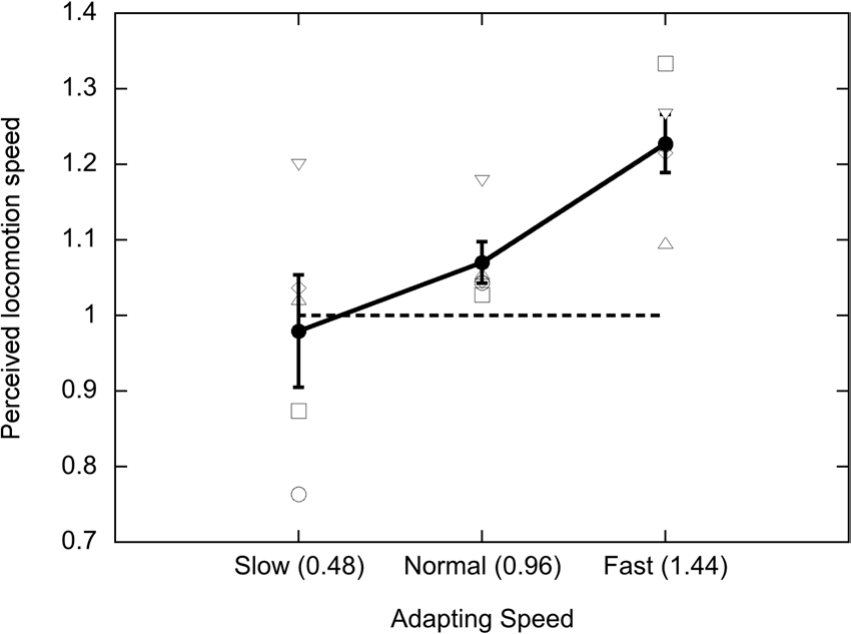
Results of Experiment 3, Apparent natural test speed as a function of adapting speed, measured using a 2AFC task. P50 points were calculated using the procedure described in Methods and Supplementary Information. Conventions as in previous figures.

Figure 3 plots the P50 as a function of the adapting speed, using the same conventions as those in Figs 1 and 2. The effect of adapting speed was significant in a one-factor ANOVA (F(2,8) = 7.22; p < 0.016; effect size η^2^_p_ = 0.64), and consistent with the effect found in Experiments 1 and 2. The same participants took part in Experiments 1 and 3, so a two-factor ANOVA was performed in order to compare the results of the two experiments, with adapting speed and psychophysical procedure as the two factors. Analysis revealed a significant main effect of adapting speed across the two experiments (F(2, 8) = 61.18; p < 0.0001; effect size η^2^_p_ = 0.939), but no main effect of procedure (F(1,4) = 4.63; p < 0.098; effect size η^2^_p_ = 0.54). The interaction between adapting speed and procedure was significant however (F(2,8) = 5.73; p < 0.029; effect size η^2^_p_ = 0.59), indicating (comparing Fig. 1 to Fig. 3) that there was a larger P50 shift in the MSS experiment than in the 2AFC experiment.

The results of Experiment 3 show that the changes in the apparent locomotion speed of PLWs found in the previous two experiments cannot be explained simply by decision factors. A significant effect of adaptation was still obtained using a bias-free 2AFC procedure. However, the P50 shift obtained using 2AFC (12.4%) is much smaller than that obtained using MSS (25.3%), so decision processing does make some contribution to the effects reported in the MSS experiments. There are also larger individual differences in the data produced by the 2AFC method than in the MSS used in previous experiments. Individual differences have also been reported in other studies using the 2AFC method^29,33,34^, though the source of these differences is unclear.

All of the P50 values in Fig. 3 are shifted towards slightly higher locomotion speeds relative to those shown in corresponding conditions of the previous experiments. For example, mean P50 after adapting to ‘normal’ speed is equal to 1.07 in Experiment 3, but was equal to 1.003 in Experiment 1 and 0.989 in Experiment 2. This implies that, according to the 2AFC data, both probe stimuli were perceived to slow down slightly after adaptation.

## Discussion

Experiment 1 involved presentation of PLW displays during both adaptation and testing (PLW-PLW), and found shifts in apparently natural test speed (P50) using the method of single stimuli (PLW-PLW / MSS). Experiment 2 measured cross-adaptation between PLWs and full-cue videos, using MSS (PLW-FCV / MSS), and found smaller P50 shifts. Experiment 3 reverted to the use of PLWs during both adaptation and testing, but used a 2AFC task (PLW-PLW, 2AFC), and again found smaller P50 shifts.

Comparisons between pairs of experiments reveal that both sensory and decision factors contributed to adaptation-induced changes in apparent locomotion speed, as follows. Experiment 1 used the same sensory stimuli as those in Experiment 3 (PLW-PLW), but invoked different decision processing (MSS vs. 2AFC). So the different results of these two experiments must be due to a change at the level of decision processing. Conversely, Experiment 1 used the same task as that in Experiment 2 (MSS), and therefore the same decision processing, but different sensory stimuli (PLW-PLW vs. PLW-FCV). So the different results of these two experiments must due to changes in sensory processing. Taken together, these pairwise comparisons show that adaptation involves both a sensory component and a decision component.

**Figure 4:**
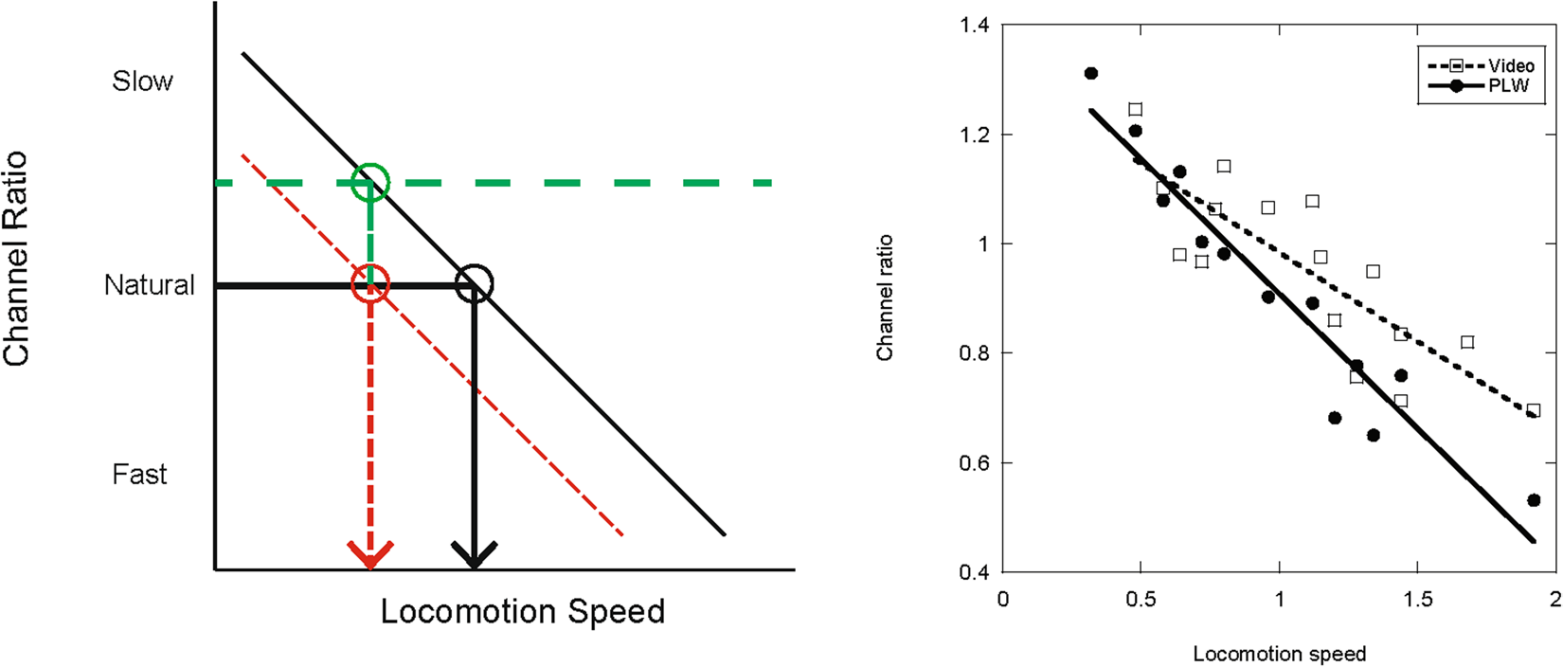
A ratio scheme for perceived locomotion speed. Left: Outline of the scheme. The thin black line shows the ratio of the outputs of two sensory channels tuned to slow and fast flicker rates, as a function of locomotion speed depicted in the stimulus. The output of slow channels declines relative to fast channels as speed increases. In decision processing, a specific criterion channel ratio is associated with ‘natural’ locomotion speed (thick black lines and circle). Sensory adaptation to relatively slow locomotion may shift the ratio line to favour the faster channel (short-dashed red lines), so that ‘natural’ locomotion corresponds to a slower stimulus speed (red circle). Decision normalisation to slow locomotion may shift the criterion line towards slower locomotion (long-dashed green line), so that ‘natural' locomotion again corresponds to a slower stimulus speed (green circle). Right: Output of a simple computational model of the ratio scheme, in which responses to stimulus displays used in the experiments were computed using physiologically plausible filters. Each filled point represents a specific PLW locomotion speed and its associated channel ratio; each open point represents a specific video locomotion speed. The solid and dashed lines show best-fitting linear functions.

Figure 4 (left) illustrates a simple conceptual scheme to explain how sensory and decision components might combine in the visual estimation of locomotion speed. Early theories of velocity constancy provide the starting point. In particular, Smith and Sherlock^35^ proposed that the stimulus correlate of apparent objective velocity is “*the frequency of objects passing a fixed point*” (p.102). In modern parlance, this stimulus property would correspond to the temporal frequency of luminance modulation in the image. Thus Burr^36^ suggested that:

“*perceived velocity is computed from the temporal frequency of signal modulation, not retinal velocity, thereby maintaining velocity constancy over a wide range of viewing conditions*.” (p.481).

So the first (sensory) stage in the proposed scheme uses a well-known velocity code that estimates speed by taking the ratio of the outputs of two classes of cortical neuron that are tuned to slow and fast rates of flicker respectively^37,38^. A simple ratio of the outputs of these two neural classes or ‘channels’ is proportional to speed, because higher speeds correspond to higher flicker rates. The thin black tilted line in Fig. 4 (left) shows how the ratio of Slow:Fast channel outputs should vary as a function of locomotion speed. The second (decision) stage in the model is to assign a specific criterion ratio as the ‘natural’ or normal speed of human locomotion, indicated by the thick black horizontal line and circle at the intersection with the ratio line. Higher channel ratios correspond to relatively slow locomotion, and lower ratios correspond to relatively fast locomotion.

In this simple norm-ratio scheme there are two ways in which an adaptation-induced change in perceived locomotion speed can arise, one at the sensory level and the other at the decision level. At the sensory level, adaptation could shift the channel ratio generated by a specific locomotion speed, due to a change in the relative responsiveness of slow and fast neurons. For example, adaptation to slow locomotion could reduce the responsiveness of ‘slow’ neurons relative to ‘fast’ neurons, biasing the ratio at any given stimulus speed in favour of the fast neurons. If the criterion level remained fixed, it would intersect the ratio line at a slower locomotion speed (red circle and short-dashed lines in Fig. 4 (left)). On the other hand, adaptation at the decision level represents a shift in the value of the criterion ratio itself. For example, after adaptation to slow locomotion, the ratio may ‘normalise’ by shifting toward values that correspond to recently experienced (slower) locomotion speeds; in other words, the criterion line would move up the vertical axis in Fig. 4 (left) so that it meets the ratio line at a value that corresponds to slower speeds (green circle and long-dashed lines in Fig. 4 (left)). The result as depicted in Fig. 4 would be a similar change in apparently natural locomotion speed to that due to sensory adaptation alone. Of course, any given shift in perceived locomotion speed could be produced by different combinations of sensory and decision adaptation. The experimental results reported earlier can be explained by proposing that both sensory and decision effects combine to produce the obtained shifts in apparent locomotion speed.

It is widely acknowledged that adaptation to visual motion has multiple sensory components^39–41^. The norm-ratio scheme presented here proposes that adaptation to locomotion speed involves a decision component as well. The sensory component of adaptation to locomotion may be mediated by V1 neurons tuned to temporal frequency. The decision component must involve a representation of how fast human figures normally move that is updated on the basis of recent experience. As discussed earlier, evidence indicates that the neural substrate for such a stored representation is likely to be located in the temporal cortex. One form of decision bias that could account for the smaller effect we obtained using 2AFC compared to MSS is the following:^42^ “*the tendency to favour one of the two response categories when observers are unsure of their answer*” (p. 186). Such a bias would shift the P50 point in the MSS experiments (in which the response categories were ‘fast’ versus ‘slow’), but not in the 2AFC experiment (in which the response categories were ‘first’ versus ‘second’ test).

Previously, the proposed ratio code for speed was tested using simple grating or bar stimuli^37,38^. The PLW and video displays used in the present experiments are much more complex and variable, so there is a major unresolved issue: Can such a simple Slow:Fast channel ratio encode the different locomotion speeds in our stimuli in the way that the scheme assumes? To address this question, a simple computational model of the ratio scheme was implemented in Matlab, using physiologically plausible ‘slow’ and ‘fast’ channels, and applied to image sequences from the PLW and video displays used in the experiments. The channel ratio was computed over the range of locomotion speeds used in the experiments. Figure 4 (right) plots channel ratio as a function of locomotion speed, for the PLW (filled symbols) and video displays (open symbols) used in the experiments. Each symbol represents the average of five randomly different animation sequences at that speed, drawn from the experimental stimuli (details of the computational modeling are available in Supplementary Information). The channel ratio varies linearly with locomotion speed, confirming that the simple norm-ratio computation in the scheme outlined in Fig. 4 (left) can provide an estimate of locomotion speed even in complex stimuli. Remarkably, roughly similar ratios are computed from PLW and video displays at similar locomotion speeds. The slope of the PLW ratio line is significantly steeper than the line for the video displays, indicating stronger differential activation of the slow and fast channels and perhaps accounting for the weaker effects obtained using videos as adapting or test displays.

It remains to be seen how stable the proposed norm-ratio measure of locomotion speed is under different viewing conditions, and using different receptive field parameters. The norm-ratio model could also be applied more generally to judging the speed of other real-world objects.

## Electronic supplementary material

Supplementary Information
