# Supplementary material for: Adaptation reveals sensory and decision components in the visual estimation of locomotion speed
Source: Sci Rep. 2018 Aug 29;8:13059. doi: 10.1038/s41598-018-30230-1 (PMC6115446; doi:10.1038/s41598-018-30230-1)
Supplement: Supplementary file 1 — Supplementary Information [file 41598_2018_30230_MOESM1_ESM.docx]

Adaptation reveals sensory and decision components in the visual estimation of locomotion speed

SUPPLEMENTARY INFORMATION

George Mather^1^ Todd Parsons^1^

1. School of Psychology, University of Lincoln, UK

Correspondence to gmather@lincoln.ac.uk

**2AFC psychophysical Procedure**

Experiment 3 used a two-alternative forced choice (2AFC) procedure to measure P50 in a way that minimises the contribution of decision factors. Following the initial period of adaptation, each probe trial involved presentation of a pair of PLW displays, drawn randomly from a set of 15 possible pairs. The locomotion speeds of the 15 pairs of displays are depicted in Figure S1.

**
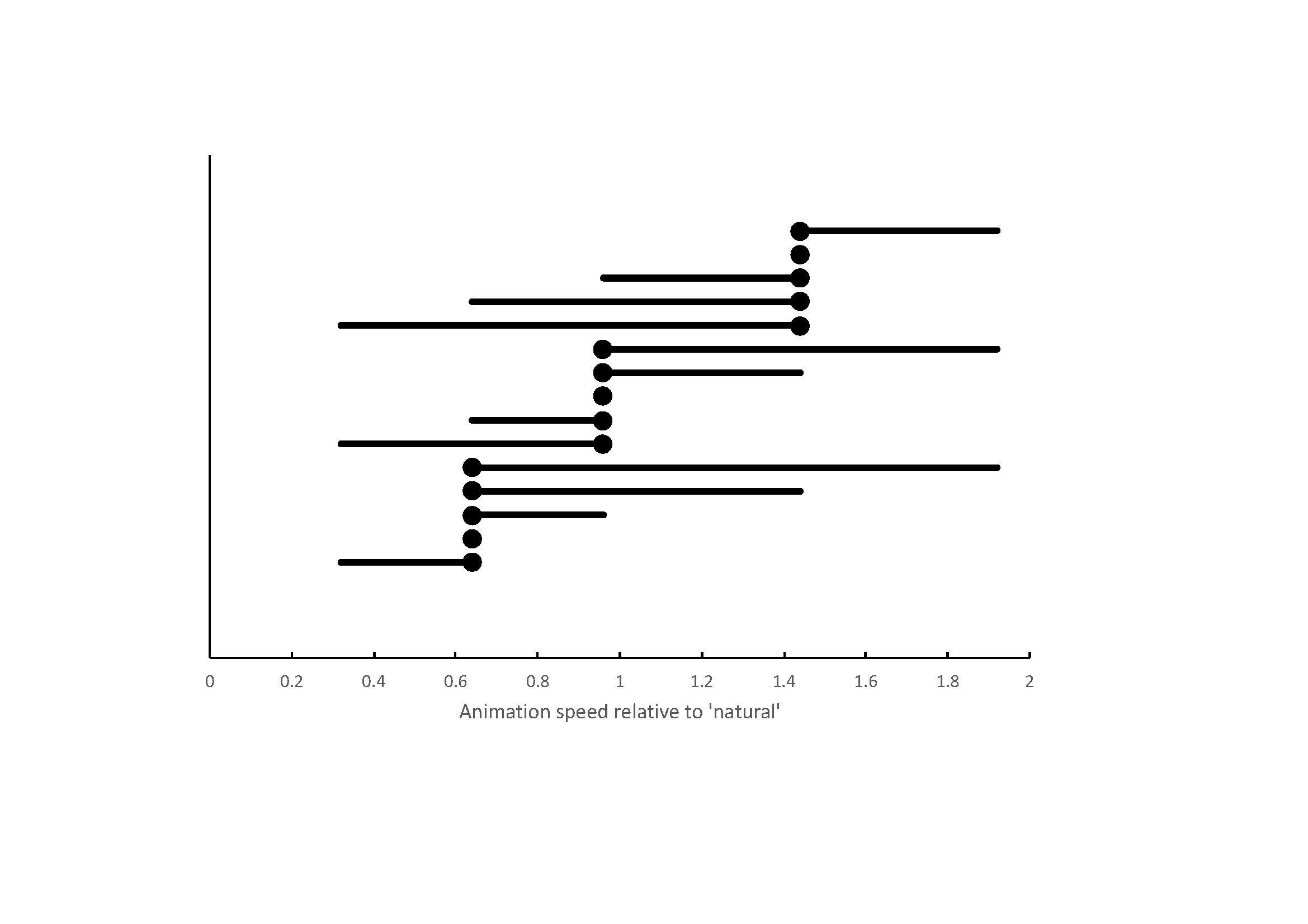
**

**Figure S1 Probe stimulus pairs used in Experiment 3**

Each line represents a specific probe stimulus pair, with the locomotion speed of each member of the pair denoted by the endpoints of the line. One member of each pair (‘pedestal’, shown by the filled circles) was assigned one of three possible speed values (0.64, 0.96, 1.44), and the other member of the pair (‘test’) was given a speed offset from this value. In six pairs the pedestal is faster than the test (circle is at the right-hand end of the line), and in six pairs the pedestal is slower than the test (circle is at the left-hand end). In the remaining three pairs, the pedestal and test had the same speeds (i.e. the test offset was zero).

Each thick horizontal line in the diagram represents a probe pair, whose locomotion speed values are denoted by the end points of the line. One member of each pair (the ‘pedestal’) was assigned one of three possible speed values (0.64, 0.96, 1.44), and the other member of each pair (the ‘test’) was given a speed offset from this pedestal so that its locomotion speed varied between 0.32x and 1.92x natural speed from trial to trial. The experiment recorded the number of probe trials in which the participant selected the pedestal display as closer to ‘natural’ speed. In six of the probe pairs, the pedestal display was faster than the test display, and in another six pairs the pedestal was slower than the test (in the remaining three stimuli the speed of the two displays was the same). The order of presentation of the two probe displays in each pair varied randomly from trial to trial. Note also that in some probe trials both displays were slower (or faster) than natural locomotion speed. So decision biases in favour of responding ‘slow’ (or ‘fast’), or towards selecting to the first (or second) display more often, or towards always selecting the slower (or faster) display more often, would not result in a shift in the P50 point because such biases would cancel out across the 15 test displays. On the other hand, sensory biases due to alterations in the response of neurons sensitive to the two probe stimuli should still produce a measurable shift in the P50.

Response data in each session were collated in terms of the proportion of responses in which the pedestal probe was selected as closer to ‘natural’ speed. In accordance with the analysis in ^1^, the following function was fitted to the data in each adapting condition:

p(‘P’) = 0.5 (1 – erf [-( *p* + *t*)/ (2σ) + µ/σ] erf[-(*p* - *t*) / (2σ) ] )

Where

p(‘P’) is the probability of the participant choosing the pedestal stimulus

*p* is the speed of the pedestal stimulus

*t* is the speed of the test stimulus

µ is the participant’s bias in their P50 value (mean of the psychometric function)

σ is the participant’s just-noticeable difference (SD of the psychometric function)

erf is the Matlab error function approximation to the cumulative normal distribution.

The best-fitting values of µ and σ were found for each participant in each adapting condition using the Matlab LSQCURVEFIT function. The value of µ corresponded to the P50 value calculated in previous experiments.

**Computational model of the norm-ratio theory of locomotion speed estimation**


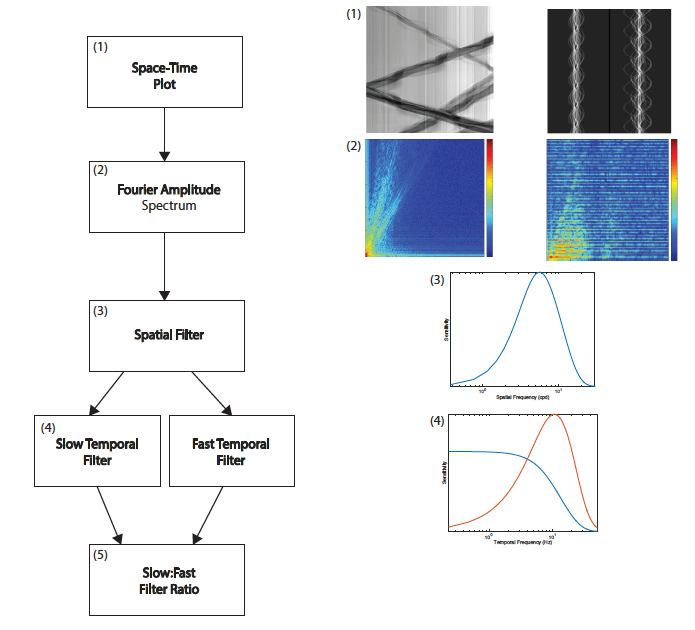


**Figure S2** Computational model of the ratio code for locomotion speed.

Left: Flow diagram showing the sequence of operations.

Right: Visualisations of example data used in the computations.

See text for details.

Figure S1 shows the five processing stages in the temporal frequency model of locomotion speed estimation, which was implemented in Matlab.

(1) Space-time plot

The process begins with the creation of a space-time plot representing the change in the input stimulus over time. The right-hand side of the Figure shows (top) example space-time plots of the video and PLW video displays. In each plot, the *x* dimension represents space, and the *y* dimension represents time (running vertically from the top). For simplicity, the model considers motion only along the horizontal axis in the animated displays, parallel to the axis of locomotion. Consider first the PLW space-time plot (top-right plot). Each row of pixels in the plot represents the horizontal luminance variation across a specific animation frame of the display (luminance is averaged along the vertical spatial axis of the frame at each horizontal position), with time (i.e. frame number) advancing in successive rows from top to bottom in the plot. The PLWs were presented in sagittal view as if walking on a treadmill, so the PLW space-time plot traces the movement of the 48 dots in the display. Each dot oscillates in its horizontal position over the step cycle, creating the multiple wave-like traces in the plot. The walkers varied in size, so the amplitude of the dot oscillations varies from dot to dot, though they all depict the same locomotion speed. The video displays contain multiple figures walking across the frame, at slightly different speeds and distances from the camera, so their space-time plots look very different. Each thick grey line in the example video space-time plot traces a specific individual advancing across the frame over time, either from left-to-right or vice-versa.

All space-time plots contained 512x512 pixels; each horizontal (spatial) pixel represented 0.0051 deg as in the experimental stimulus; each vertical (temporal) pixel represented a time interval of (1/120 * FD), where FD represents the duration of each animation frame in seconds (FD varied in different stimuli).

(2) Fourier spectrum

Matlab’s FFT2 function is used to compute the Fourier magnitude spectrum of each space-time plot. Figure S1 depicts the positive quadrant of the PLW or video FFT in terms of log magnitude. The origin at bottom-left corresponds to zero frequency, with increasing spatial frequency plotted along the horizontal axis and temporal frequency along the vertical axis. Magnitude is colour-coded. The speed signal corresponds to the oriented spatiotemporal energy in the plot.

(3) Spatial filtering

Next, the Fourier spectrum is filtered in the spatial frequency dimension (*f*), according to the Fourier domain spatial frequency tuning profile of the filters. The spatial frequency tuning function is a based on a balanced difference-of-Gaussians receptive field profile, defined in ^2^ as follows:

dog(*f*) = √ π σ_c_ exp(-π^2^ *f*^2^ σ_c_^2^) – k √ π σ_s_ exp(-π^2^ *f*^2^ σ_s_^2^)

Where:

σ_c_ = Space constant of excitatory centre

σ_s_ = Space constant of inhibitory surround

k = balance factor between excitation and inhibition.

The values for these parameters were taken from Hawken and Parker’s best-fitting estimates for receptive fields in macaque V1: σ_c_ = 1.71 arc min; σ_s_ = 5.49 arc min; k = (σ_c_/σ_s_).

Figure S1 includes a plot of this tuning function.

(4) Temporal filtering

The spatially filtered Fourier spectrum is then filtered in the temporal frequency dimension (*ω*) to create the outputs of two different temporal filters, one slow (p) and the other fast (m). The temporal tuning functions are based on estimates of the temporal tuning of V1 neurons, as defined in ^3^:

p(*ω*) = √(a^2^ + b^2^)

m(*ω*) = (*ω/k)* p(*ω*)

Where

a = ((2 π *ω* *τ*_1_)^2^ + 1)^−9/2^

b = ((2 π *ω* *τ*_2_)^2^ + 1)^−10/2^

and

*τ*_1_ = 0.0072

*τ*_2_ = 0.0043

*k* = 4.0

For simplicity, the sequential spatial and temporal filtering operations used here assume spatiotemporal separability, as discussed in ^3^.

(5) Filter ratio computation

The ratio of the summed outputs of the two filters is computed to provide a summary statistic of the ratio computation for the space-time image.

**References**

1. Morgan, M. J., Schreiber, K. & Solomon, J. A. Low-level mediation of directionally specific motion aftereffects: Motion perception is not necessary. *Attention, Perception, Psychophys.* (2016). doi:10.3758/s13414-016-1160-1

2. Hawken, M. J. & Parker, a. J. Spatial Properties of Neurons in the Monkey Striate Cortex. *Proc. R. Soc. B Biol. Sci.* **231,** 251–288 (1987).

3. Perrone, J. Economy of scale: A motion sensor with variable speed tuning. *J. Vis.* **5,** 28–33 (2005).
